# Baseline neutrophil-to-lymphocyte ratio combined with SLEDAI predicts lupus low disease activity state at 1 year: a cohort study

**DOI:** 10.3389/fimmu.2026.1798433

**Published:** 2026-06-15

**Authors:** Yadan Zou, Ruohan Yu, Kun Yang, Lina Zhang, Jing Zhang, Ji Li, Ting Long, Yanfeng Zhang, Sheng-Guang Li

**Affiliations:** 1 Department of Rheumatology and Immunology, Peking University International Hospital, Beijing, China; 2 School of Clinical Medicine, Shanxi Medical University, Taiyuan, China

**Keywords:** lupus, Systemic, neutrophil-to-lymphocyte ratio, low disease activity state, cohort Study

## Abstract

**Background:**

Systemic lupus erythematosus (SLE) is a heterogeneous autoimmune disease in which treat-to-target management increasingly emphasizes attainment of Lupus Low Disease Activity State (LLDAS). Complete blood count (CBC)-derived inflammatory indices are inexpensive and readily available markers, but their value for predicting subsequent LLDAS remains unclear.

**Methods:**

This retrospective cohort study included 165 hospitalized patients with SLE at Peking University International Hospital between 2022 and 2024 who had baseline CBC data and completed 1-year follow-up. Baseline neutrophil-to-lymphocyte ratio (NLR), platelet-to-lymphocyte ratio (PLR), monocyte-to-lymphocyte ratio (MLR), systemic immune-inflammation index (SII), and systemic inflammation response index (SIRI) were calculated from routine hematologic parameters. The primary outcome was LLDAS achievement at 1 year according to Asia Pacific Lupus Collaboration criteria. Univariable and multivariable logistic regression analyses were performed. Multivariable models adjusted for baseline SLEDAI-2K, age, sex, immunosuppressant use, and prednisone dose. Receiver operating characteristic (ROC) analysis, bootstrap internal validation, calibration, and decision curve analysis (DCA) were also conducted.

**Results:**

Compared with non-LLDAS patients, those who achieved LLDAS had lower baseline SLEDAI-2K, lower prevalence of renal involvement, lower neutrophil counts, higher hemoglobin levels, and lower baseline NLR, PLR, SII, and SIRI. In univariable analysis, higher baseline NLR (OR 0.69, 95% CI 0.57-0.83, *P* < 0.001), PLR (OR 0.96 per 10-unit increase, 95% CI 0.93-0.99, *P* = 0.008), and SII (OR 0.86 per 100-unit increase, 95% CI 0.79-0.94, *P* < 0.001) were associated with lower odds of LLDAS. In the adjusted model, baseline NLR remained independently associated with LLDAS attainment (aOR 0.690, 95% CI 0.561-0.848, *P* < 0.001). The NLR+SLEDAI-2K model achieved an AUC of 0.746, with limited optimism on bootstrap validation.

**Conclusion:**

Baseline CBC-derived inflammatory indices, particularly NLR, were associated with 1-year LLDAS attainment in SLE. NLR, combined with SLEDAI-2K, may provide a practical, low-cost adjunct for early risk stratification in treat-to-target-oriented SLE management.

## 1 Introduction

Systemic lupus erythematosus (SLE) is a chronic autoimmune disease characterized by marked clinical and immunological heterogeneity and an unpredictable relapsing–remitting course. The global burden remains substantial, with an estimated incidence of 5.14 per 100–000 person-years and a prevalence of about 3.41 million worldwide (1). Recurrent flares and persistent inflammation drive irreversible organ damage, impair quality of life, and increase health-care utilization, creating a need for monitoring tools that are repeatable, low cost, and available at every outpatient visit.

In routine practice, disease activity is commonly assessed using validated indices—most often the Systemic Lupus Erythematosus Disease Activity Index 2000 (SLEDAI-2K)—together with serological markers such as complement and anti–double-stranded DNA (anti-dsDNA) (2). Complement testing is widely used to support assessment of immunological activity, consistent with the role of complement dysregulation in SLE (3). However, serologies can be discordant with symptoms and organ involvement in individual patients, and testing frequency, turnaround time, and cost vary across care settings—highlighting the need for complementary, readily obtainable markers.

Treat-to-target (T2T) has therefore emerged as a pragmatic framework for SLE management, prioritizing remission when achievable, or otherwise the lowest feasible disease activity with minimal treatment toxicity (4). To operationalize T2T, the Asia Pacific Lupus Collaboration proposed the Lupus Low Disease Activity State (LLDAS), integrating both disease activity and treatment domains (5) Achieving LLDAS for at least 50% of follow-up has been associated with reduced damage accrual and lower mortality (6). However, many patients fail to reach LLDAS within a defined period, underscoring the need for early, practical indicators that can inform treatment intensity and monitoring frequency.

A scalable approach is to use inflammation-related indices derived from the routine complete blood count (CBC), including the neutrophil-to-lymphocyte ratio (NLR) and platelet-to-lymphocyte ratio (PLR). These measures are cost-free, easily repeatable, and may reflect the balance between innate immune activation and adaptive immune suppression. A systematic review and meta-analysis supports the diagnostic performance of NLR and PLR in SLE (7), and prospective data suggest that these indices correlate with disease activity and flares (8). However, the majority of existing studies are cross-sectional in design, providing evidence for association rather than prediction. Consequently, evidence remains limited on whether baseline CBC-derived indices can predict attainment of a treat-to-target (T2T) endpoint, particularly the Lupus Low Disease Activity State (LLDAS), in a longitudinal manner. To address this gap, we designed a retrospective cohort study with 1−year follow−up. This study aimed to determine whether baseline indices independently predict LLDAS attainment during follow−up after comprehensive adjustment for potential confounders (including glucocorticoid and immunosuppressant use), thereby providing a practical, longitudinal adjunct for T2T-oriented care.

## 2 Methods

### 2.1 Study design and participants

This retrospective cohort study included 165 patients with systemic lupus erythematosus (SLE) who were hospitalized at Peking University International Hospital between 2022 and 2024, had baseline complete blood count (CBC) data available, and completed a 1-year follow-up assessment. Baseline demographic data, clinical characteristics, and laboratory measurements were collected at enrollment. Baseline demographic and clinical data collected included age, sex, disease duration, body mass index (BMI), organ involvement (renal, neurologic, hematologic, mucocutaneous, musculoskeletal, serositis), autoantibody profiles (anti-dsDNA, low C3, low C4, IgG, IgA, IgM), and laboratory parameters (complete blood count, serum creatinine). SLEDAI-2K was calculated using clinical and laboratory information available at the baseline. Baseline treatments were recorded, including prednisone use and initial daily dose (mg/day prednisone equivalent), hydroxychloroquine, any immunosuppressant use (as well as specific agents: mycophenolate mofetil, cyclophosphamide, azathioprine, calcineurin inhibitors), and biologic agents. Patients were included if they fulfilled ACR 1997 or EULAR/ACR 2019 classification criteria for SLE and had complete information required to evaluate the primary outcome at 1 year. The exclusion criteria were as follows: (1) active malignancy; (2) severe concurrent infection at baseline; (3) history of hematologic malignancies.

The study was approved by the institutional ethics committee, which waived the need for patient written informed consent because this was a retrospective study.

### 2.2 CBC-derived inflammatory indices

CBC-derived inflammatory indices were calculated at baseline using absolute cell counts and platelet counts obtained from routine hematology testing:

1. Neutrophil-to-lymphocyte ratio (NLR) = absolute neutrophil count/absolute lymphocyte count
2. Platelet-to-lymphocyte ratio (PLR) = platelet count/absolute lymphocyte count
3. Monocyte-to-lymphocyte ratio (MLR) = absolute monocyte count/absolute lymphocyte count
4. Systemic immune-inflammation index (SII) (9) = platelet count × absolute neutrophil count/absolute lymphocyte count
5. Systemic inflammation response index (7-12SIRI) (10) = absolute neutrophil count × absolute monocyte count/absolute lymphocyte count

### 2.3 Outcome definition: LLDAS at 1-year follow-up

The primary outcome was achievement of lupus low disease activity state (LLDAS) (5) at the 1-year follow-up visit, defined according to the standard Asia Pacific Lupus Collaboration (APLC) criteria: (1) SLEDAI-2K ≤4 with no activity in major organ systems (renal, CNS, cardiopulmonary, vasculitis, fever) and no hemolytic anemia or gastrointestinal activity; (2) no new features of lupus disease activity compared with the previous assessment; (3) Physician Global Assessment (PGA) ≤1 (0–3 scale); (4) prednisolone (or equivalent) dose ≤7.5 mg/day; and (5) well-tolerated standard maintenance doses of immunosuppressive drugs and approved biologics, excluding investigational agents.

### 2.4 Statistical analysis

Continuous variables are presented as mean ± standard error (SE) or median [interquartile range] depending on distribution, and categorical variables as number (percentage). Baseline characteristics were compared between LLDAS and non-LLDAS groups using independent-samples t-test or Mann–Whitney U test for continuous variables, and chi-square or Fisher’s exact test for categorical variables.

Univariable logistic regression was performed to examine associations of each variable with LLDAS attainment. To improve clinical interpretability, certain variables were scaled: platelet-to-lymphocyte ratio (PLR) per 10-unit increase, systemic immune-inflammation index (SII) per 100-unit increase, and monocyte-to-lymphocyte ratio (MLR) per 0.1-unit increase. Neutrophil-to-lymphocyte ratio (NLR), systemic inflammation response index (SIRI), and SLEDAI-2K were analyzed per 1-unit increase.

For multivariable analysis, a core logistic regression model was constructed including the following prespecified covariates: baseline NLR (per 1-unit), baseline SLEDAI-2K (per 1-point), age, sex, baseline immunosuppressant use (yes/no), and baseline prednisone dose (continuous, mg/day prednisone equivalent). Adjusted odds ratios (aOR) with 95% confidence intervals (CI) were reported. Multicollinearity was assessed using variance inflation factor (VIF), with VIF <5 considered acceptable.

To evaluate the robustness of the association between NLR and LLDAS, three sensitivity analyses were performed: Model 1 excluded all treatment variables (immunosuppressant use and prednisone dose); Model 2 additionally included change in SLEDAI from baseline to follow-up (ΔSLEDAI); Model 3 replaced continuous prednisone dose with a three-category glucocorticoid variable (low <7.5 mg/day, moderate 7.5–30 mg/day, high >30 mg/day).

Discrimination performance was assessed using receiver operating characteristic (ROC) curves and the area under the curve (AUC). For combined models, predicted probabilities were derived from logistic regression. Internal validation was performed using bootstrap resampling with 1,000 iterations according to Harrell’s method to estimate optimism and calculate optimism-corrected AUC for both the 2-variable (NLR+SLEDAI) and full multivariable models.

Calibration curves were plotted using locally weighted regression (LOESS) with 500 bootstrap resamples to assess agreement between predicted and observed probabilities. Brier scores and scaled Brier scores were calculated. Decision curve analysis (DCA) was performed to evaluate the clinical net benefit of the models across a range of threshold probabilities.

All analyses were performed using R-4.2.1 (R Foundation for Statistical Computing, Vienna, Austria) and Free Statistics software version 2.0 (Beijing Free Clinical Medical Technology Co., Ltd.). All statistical tests, including chi-square tests, were conducted as two-sided with a significance level of 0.05.

## 3 Results

### 3.1 Baseline demographic, clinical, serologic, and treatment characteristics of the cohort overall and according to 1-year LLDAS status

The baseline demographic, clinical, serologic, and treatment characteristics of the 165 SLE patients are summarized in **Table 1**, stratified by LLDAS attainment at 1 year.

**Table 1:** Baseline demographic, clinical, serologic, and treatment characteristics of the cohort overall and according to 1-year LLDAS status.

Characteristic	Overall (N = 165)	LLDAS at 1 year (n=55)	Non-LLDAS at 1 year (n=110)	*P* value
Age, years	43.6 ± 1.20	43.31 ± 1.98	43.82 ± 1.51	0.921
Sex				
Male	28	4	24	0.019
Female	137	51	86	
Disease duration, years	8.45 ± 0.588	7.13 ± 0.98	9.11 ± 0.73	0.082
Baseline SLEDAI	6.81 ± 0.38	5.53 ± 0.62	7.86 ± 0.46	<0.001
Skin and hair involvement	35 (21.2%)	9 (16.4%)	26 (23.6%)	0.281
Serositis	9 (5.5%)	4(7.3%)	5 (4.5%)	0.483
Musculoskeletal involvement	17 (10.3%)	3 (5.5%)	14 (12.7%)	0.182
Haematological involvement	43 (26.1%)	14 (25.5%)	29 (26.4%)	0.900
Renal involvement	81 (49.1%)	19(34.5%)	62(56.4%)	0.008
Central nervous system involvement	6 (3.6%)	0(0%)	6 (5.5%)	0.180
Anti-dsDNA	253.15 ± 23.20	216.60 ± 35.62	279.91 ± 29.12	0.386
Low C3	106 (64.2%)	33 (60.0%)	73 (66.4%)	0.421
Low C4	44 (26.7%)	12 (21.8%)	32(29.1%)	0.319
IgG	12.07 ± 0.46	12.80 ± 0.80	11.92 ± 0.59	0.296
IgA	3.15 ± 0.22	3.41 ± 0.35	2.98 ± 0.26	0.049
IgM	1.04 ± 0.07	1.11 ± 0.11	1.01 ± 0.08	0.136
WBC(Í10^9^/L)	5.62 ± 0.22	5.10 ± 0.29	5.92 ± 0.29	0.143
Hemoglobin(g/L)	114.11 ± 1.54	119.69 ± 2.43	110.01 ± 1.93	<0.001
Platelet(*10^9^/L)	199.95 ± 5.60	203.19 ± 9.21	201.05 ± 7.18	0.924
Monocyte(*10^9^/L)	0.42 ± 0.03	0.47 ± 0.08	0.40 ± 0.03	0.584
Neutrophil(*10^9^/L)	3.99 ± 0.18	3.43 ± 0.26	4.28 ± 0.23	0.030
Lymphocyte(*10^9^/L)	1.20 ± 0.06	1.31 ± 0.08	1.15 ± 0.08	0.008
NLR	4.16 ± 2.89	2.99 ± 0.27	5.12 ± 0.39	<0.001
PLR	224.17 ± 155.54	180.04 ± 11.71	260.64 ± 22.11	0.035
MLR	0.42 ± 0.37	0.40 ± 0.07	0.43 ± 0.03	0.153
SII	832.09 ± 664.38	613.50 ± 65.12	1017.25 ± 86.22	0.001
SIRI	1.60 ± 1.42	1.53 ± 0.40	1.82 ± 0.15	0.011
Serum creatinine(μmol/L)	125.37 ± 11.38	105.58 ± 19.17	135.26 ± 14.09	0.004
Prednisone use	156 (94.5%)	48 (87.3%)	108 (98.2%)	0.007
Prednisone initial dose, mg/day	10.00(25.00)	29.12(16.25)	15.00(22.50)	<0.001
Hydroxychloroquine use	135 (81.8%)	49 (89.1%)	86 (78.2%)	0.087
Any immunosuppressant use	124 (75.2%)	41 (74.5%)	83 (75.5%)	0.899
Biologic use	67 (40.6%)	21 (38.2%)	46 (41.8%)	0.654
MMF use	61 (37.0%)	20 (36.4%)	41 (37.3%)	0.909
Cyclophosphamide use	11 (6.7%)	2 (3.6%)	9 (8.2%)	0.270
Azathioprine use	14 (8.5%)	5 (9.1%)	9 (8.2%)	0.843
Calcineurin inhibitor use	35 (21.2%)	9 (16.4%)	26 (23.6%)	0.281

LLDAS, lupus low disease activity state; SLEDAI, Systemic Lupus Erythematosus Disease Activity Index; anti-dsDNA, anti-double-stranded DNA antibody; C3/C4, complement C3/C4; IgG/IgA/IgM, immunoglobulin G/A/M; WBC, white blood cell count; NLR, neutrophil-to-lymphocyte ratio; PLR, platelet-to-lymphocyte ratio; MLR, monocyte-to-lymphocyte ratio; SII, systemic immune-inflammation index; SIRI, systemic inflammation response index; MMF, mycophenolate mofetil. Data are presented as n (%) or mean ± SE / median (IQR), as appropriate. Percentages for LLDAS and non-LLDAS groups were recalculated using the corresponding column denominators (n=55 and n=110).

Compared with the non-LLDAS group, the LLDAS group had a lower proportion of males (7.3% vs. 21.8%, *P* = 0.019), lower baseline SLEDAI-2K (5.53 ± 0.62 vs. 7.86 ± 0.46, *P* < 0.001), and lower prevalence of renal involvement (34.5% vs. 56.4%, *P* = 0.008). Hemoglobin levels were higher in the LLDAS group (119.69 ± 2.43 vs. 110.01 ± 1.93 g/L, *P* < 0.001), while neutrophil counts were lower (3.43 ± 0.26 vs. 4.28 ± 0.23 ×10^9^/L, *P* = 0.030). All CBC-derived inflammatory indices except MLR were significantly lower in patients who later achieved LLDAS (all *P* < 0.05). Baseline prednisone dose was lower in the LLDAS group (median [IQR] shown in **Table 1**). Immunosuppressant use did not differ between groups (*P* = 0.899).

### 3.2 Logistic regression analyses for predictors of LLDAS

Univariable logistic regression analyses (**Table 2**) showed that higher baseline NLR (crude OR 0.69 per 1-unit, 95% CI 0.57–0.83, *P* < 0.001), PLR (OR 0.96 per 10-unit, 95% CI 0.93–0.99, *P* = 0.008), and SII (OR 0.86 per 100-unit, 95% CI 0.79–0.94, *P* < 0.001) were significantly associated with lower odds of achieving LLDAS. SIRI and MLR were not significant. Among demographic variables, female sex was associated with higher odds of LLDAS (OR 4.18, 95% CI 1.19–14.65, *P* = 0.025). Glucocorticoid category (ordinal) was strongly associated with LLDAS (OR 0.33, 95% CI 0.19–0.58, *P* < 0.001), while immunosuppressant and hydroxychloroquine use were not (**Table 2**).

**Table 2:** Univariable logistic regression: predictors of LLDAS achievement at 1 year.

Variable	Crude OR	95% CI	*P*-value
Demographics			
Age (per 1-year increase)	1	0.98–1.02	0.828
Female	4.18	1.19–14.65	0.025
BMI (per 1 kg/m²)	0.95	0.86–1.04	0.278
Inflammatory Indices			
NLR (per 1-unit increase)	0.69	0.57–0.83	<0.001
PLR (per 10-unit increase)	0.96	0.93–0.99	0.008
SII (per 100-unit increase)	0.86	0.79–0.94	<0.001
SIRI (per 1-unit increase)	0.91	0.74–1.13	0.394
MLR (per 0.1-unit increase)	0.99	0.90–1.09	0.775
Disease Activity			
SLEDAI-2K (per 1-unit increase)	0.87	0.80–0.95	0.002
Treatment			
Immunosuppressant use	0.99	0.46–2.11	0.971
Hydroxychloroquine use	2.07	0.79–5.43	0.139
Glucocorticoid category (ordinal)	0.33	0.19–0.58	<0.001

LLDAS, lupus low disease activity state; NLR, neutrophil−to−lymphocyte ratio; PLR, platelet−to−lymphocyte ratio; SII, systemic immune−inflammation index; SIRI, systemic inflammation response index; MLR, monocyte−to−lymphocyte ratio; SLEDAI−2K, Systemic Lupus Erythematosus Disease Activity Index 2000; BMI, body mass index.

In the multivariable logistic regression model adjusting for age, sex, baseline SLEDAI-2K, immunosuppressant use, and continuous prednisone dose (**Table 3**, main model), baseline NLR remained independently associated with LLDAS attainment (aOR 0.690, 95% CI 0.561–0.848, *P* < 0.001). Baseline SLEDAI-2K (aOR 0.872, 95% CI 0.790–0.961, *P* = 0.006) and female sex (aOR 4.583, 95% CI 1.178–17.830, *P* = 0.028) were also independent predictors. Immunosuppressant use and prednisone dose were not significant in the multivariable model. All variance inflation factors were <5, indicating no problematic multicollinearity.

**Table 3:** Multivariable logistic regression: adjusted odds ratios for LLDAS achievement at 1 year.

Variable	Adjusted OR	95% CI	*P-*value
NLR (baseline, per 1-unit)	0.69	0.561–0.848	<0.001
SLEDAI-2K (baseline, per 1-point)	0.872	0.790–0.961	0.006
Age (per 1-year)	0.996	0.971–1.021	0.728
Female	4.583	1.178–17.830	0.028
Immunosuppressant use (yes vs. no)	1.243	0.521–2.965	0.623
Prednisone dose (per 1 mg/day, continuous)	1.003	0.999–1.007	0.143
Sensitivity analyses			
NLR — Model 1 (without treatment variables)	0.714	0.589–0.866	0.001
NLR — Model 2 (including ΔSLEDAI†)	0.645	0.506–0.822	<0.001
NLR — Model 3 (using GC as 3-category‡)	0.73	0.600–0.890	0.002

aOR, adjusted odds ratio; CI, confidence interval; GC, glucocorticoid.

Main model adjusted for baseline NLR, SLEDAI−2K, age, sex, immunosuppressant use, and continuous prednisone dose.

^†^ΔSLEDAI = follow-up SLEDAI minus baseline SLEDAI.

^‡^GC categories: low (<7.5 mg/day), moderate (7.5–30 mg/day), high (>30 mg/day).

All variance inflation factors were <5, indicating no significant multicollinearity. Model fit for main model: AIC = 178.0, McFadden pseudo-R²=0.188. EPV = 51 events ÷ 6 predictors = 8.5. N = 162 due to missing prednisone dose data in 3 patients.

### 3.3 Sensitivity analyses

Sensitivity analyses confirmed the robustness of the NLR effect (**Table 3**, lower section): when treatment variables were omitted (Model 1), NLR aOR was 0.714 (*P* = 0.001); after additionally adjusting for ΔSLEDAI (Model 2), NLR aOR was 0.645 (*P* < 0.001); and when continuous prednisone dose was replaced by three-category glucocorticoid (Model 3), NLR aOR was 0.730 (*P* = 0.002).

### 3.4 Model performance, internal validation, and decision curve analysis

**Figure 1:**
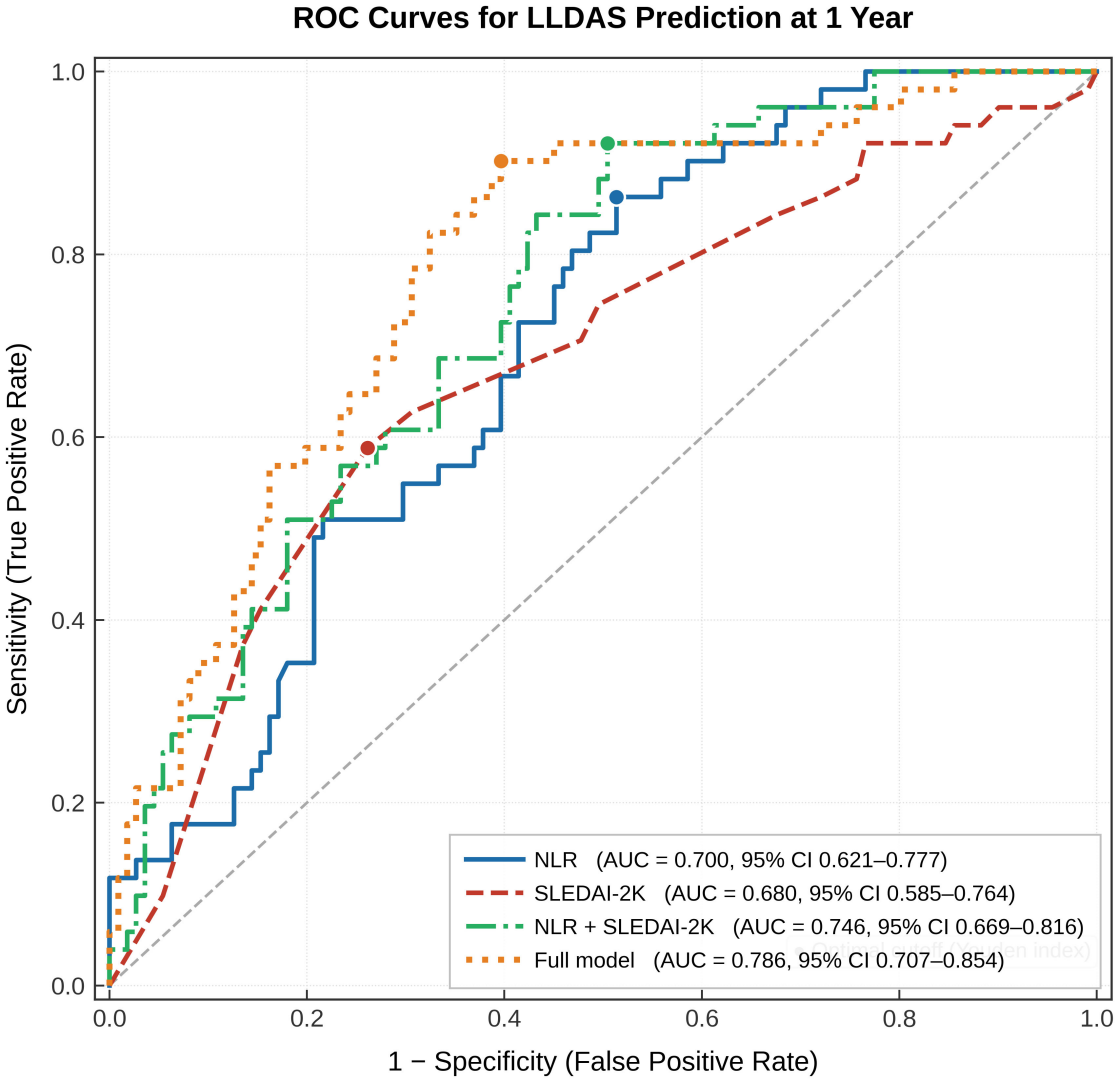
Receiver operating characteristic (ROC) curves of baseline NLR, baseline SLEDAI-2K, and combined models for predicting LLDAS achievement at 1-year follow-up. ROC curves were generated for four predictors: baseline NLR alone, baseline SLEDAI-2K alone, a 2-variable model combining NLR and SLEDAI-2K, and a full multivariable model incorporating NLR, SLEDAI-2K, age, sex, immunosuppressant use, and prednisone dose. Filled circles indicate the optimal operating point for each model, determined by the maximum Youden index (sensitivity + specificity − 1). The dashed diagonal line represents chance-level discrimination (AUC = 0.500). All 95% confidence intervals were derived from 1000-iteration bootstrap resampling. AUC, area under the curve; CI, confidence interval; LLDAS, lupus low disease activity state; NLR, neutrophil-to-lymphocyte ratio; SLEDAI-2K, Systemic Lupus Erythematosus Disease Activity Index 2000.

ROC analysis (**Figure 1**) showed that baseline NLR alone achieved an AUC of 0.700 (95% bootstrap CI 0.621–0.777) with an optimal cutoff of 3.92 (sensitivity 86.3%, specificity 48.6%). Baseline SLEDAI−2K yielded an AUC of 0.680 (0.585–0.764). The combined NLR+SLEDAI model (2−variable) improved discrimination to an AUC of 0.746 (0.669–0.816). The full multivariable model (6 covariates) achieved an AUC of 0.786 (0.707–0.854).

To assess overfitting, we performed bootstrap internal validation with 1,000 resamples (**Table 4**). For the 2−variable model (NLR+SLEDAI), the optimism−corrected AUC was 0.740 (original AUC 0.746, optimism 0.007). For the full 6−variable model, the optimism−corrected AUC was 0.754 (original AUC 0.786, optimism 0.032). These results indicate minimal overfitting and support the stability of both models.

**Table 4:** Internal validation of predictive models for LLDAS achievement using bootstrap resampling (1,000 iterations).

Model	Covariates	Original AUC	Mean optimism	Optimism-corrected AUC
NLR+ SLEDAI-2K (2-variable)	NLR, SLEDAI-2K	0.746	0.007	0.740
Full multivariable model (6-variable)	NLR,SLEDAI-2K, age, sex, immunosuppressant, prednisone (continuous)	0.786	0.032	0.754

AUC, area under the receiver operating characteristic curve; LLDAS, lupus low disease activity state; NLR, neutrophil-to-lymphocyte ratio; SLEDAI-2K, Systemic Lupus Erythematosus Disease Activity Index 2000.

**Figure 2:**
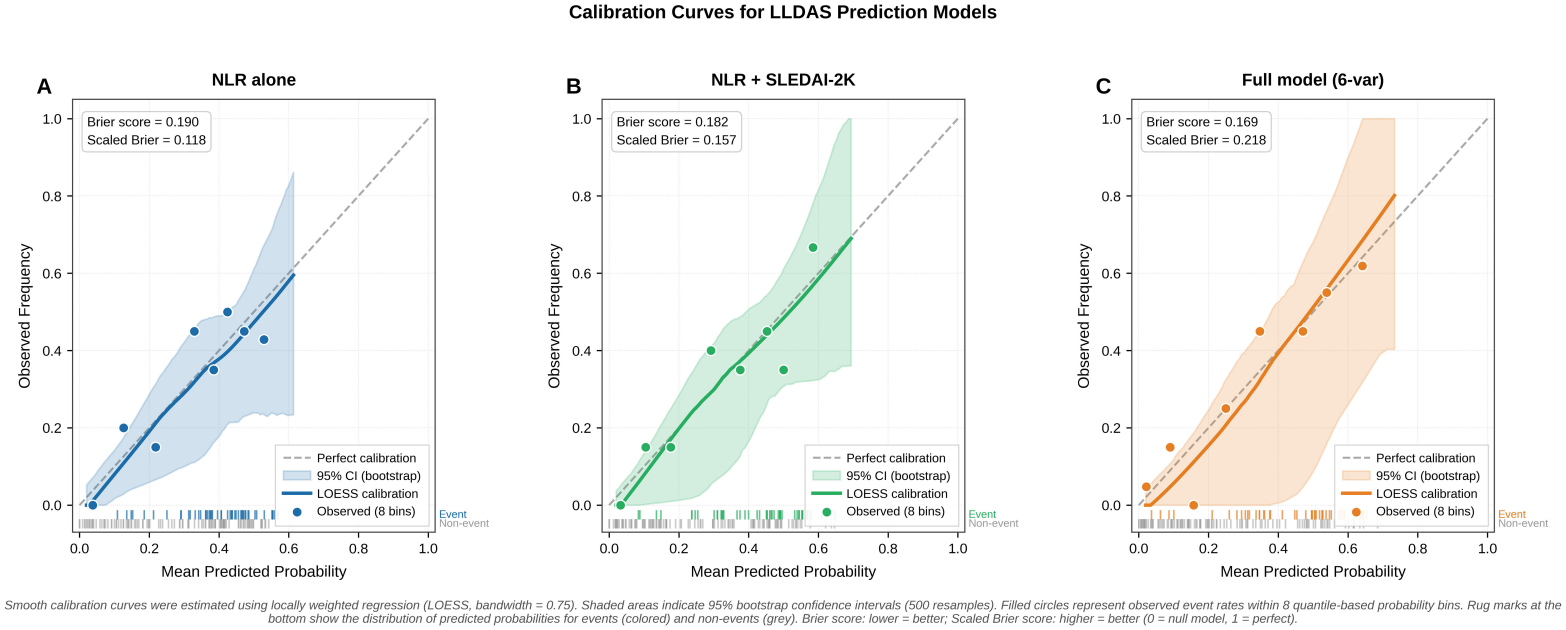
Calibration curves for the three LLDAS prediction models. **(A)** Calibration plot for the NLR‑alone model; **(B)** NLR+SLEDAI‑2K model; **(C)** full six‑variable model.

**Figure 3:**
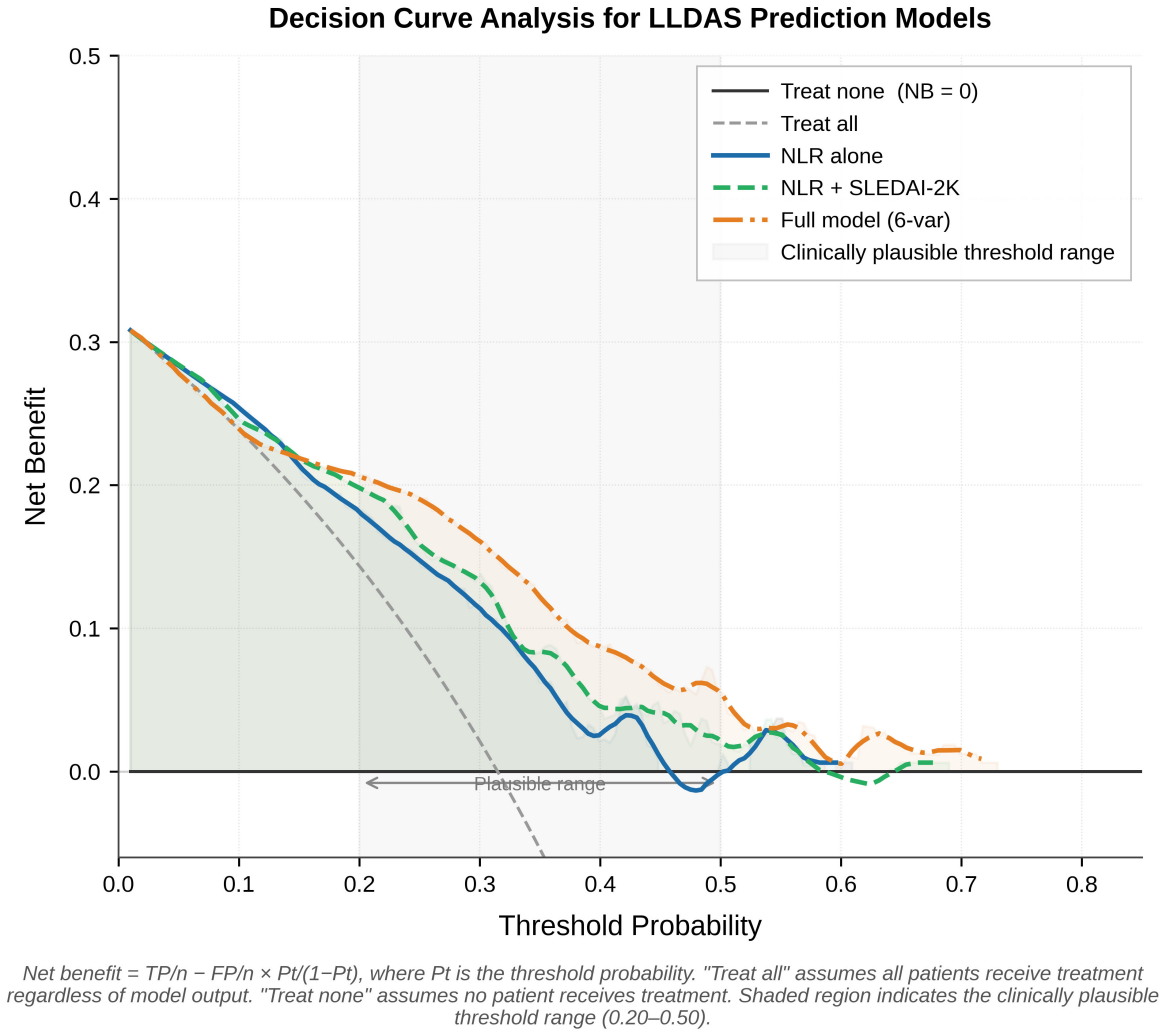
Decision curve analysis for the three LLDAS prediction models. Net benefit is plotted against threshold probability. The "Treat all" and "Treat none" strategies serve as reference lines. Shaded region (0.20–0.50) indicates the clinically plausible threshold range for initiating treatment escalation.

Calibration curves (**Figure 2**) demonstrated good agreement between predicted and observed probabilities for all models; the full model had the lowest Brier score (0.169) and a scaled Brier score of 0.218. Decision curve analysis (**Figure 3**) showed that the full model provided positive net benefit across a wide range of threshold probabilities (0.10–0.60), and the 2-variable model achieved comparable net benefit in the low-to-moderate threshold range (0.20–0.40).

## 4 Discussion

This study provides practical, longitudinal evidence that CBC-derived inflammatory indices may help inform treat-to-target (T2T) management in SLE. Unlike most previous cross-sectional studies, our retrospective cohort design with 1-year follow-up allowed assessment of longitudinal prediction rather than concurrent association alone. Patients who achieved LLDAS at 1 year had significantly lower baseline NLR, PLR, SII, and SIRI. After adjustment for baseline disease activity, age, sex, glucocorticoid dose, and immunosuppressant use, baseline NLR remained independently associated with LLDAS attainment. Furthermore, bootstrap internal validation suggested limited optimism, and decision curve analysis supported potential clinical net benefit across relevant threshold ranges. Taken together, these findings suggest that combining a simple hematologic marker with a standard disease activity index may provide a practical approach to early risk stratification, rather than a standalone prediction tool (5, 6, 11).

Our results align with prior evidence linking NLR and PLR to SLE disease activity and inflammatory burden. Meta-analyses have confirmed that NLR and PLR are higher in SLE and lupus nephritis, and correlate with SLEDAI (7, 12). Several studies show that elevated NLR predicts flares and adverse outcomes, supporting its role as a dynamic marker of systemic inflammation (13, 14). Unlike most studies focusing on disease activity or organ-specific outcomes, our work centers on LLDAS—a validated, actionable T2T endpoint that integrates disease control and treatment intensity (4–6). We also demonstrated that combining inflammatory indices with a clinical score improves discrimination, and bootstrap internal validation confirmed minimal overfitting. Decision curve analysis further supported the clinical net benefit of these models, mirroring real-world outpatient decision-making.

Mechanistically, increased NLR reflects enhanced neutrophil-driven innate immunity and relative lymphopenia, both hallmarks of active SLE. Neutrophils contribute to immune complex formation, NETs release, and type I interferon amplification (15, 16). Conversely, lymphopenia marks adaptive immune dysfunction and higher disease activity (17). These abnormalities occur alongside complement activation, which modulates immune complex clearance and tissue injury (3, 18). However, NLR is not entirely disease-specific; glucocorticoids, infection, or hematologic involvement can alter its value, emphasizing the need for contextual interpretation and sensitivity analyses (19, 20).

Clinically, our findings suggest that the simpler 2-variable model (NLR + SLEDAI-2K) may serve as a low-cost and readily available adjunct for identifying patients less likely to achieve LLDAS, potentially supporting closer follow-up and earlier treatment optimization. This model achieved an optimism-corrected AUC of 0.740 and showed comparable net benefit to the full model in the low-to-moderate threshold range (0.20-0.40). When additional clinical information is available, the full 6-variable model may offer modestly better discrimination (optimism-corrected AUC 0.754) and positive net benefit across a wider threshold range (0.10-0.60). Both approaches should be viewed as complementary to, rather than replacements for, established serological markers such as complement and anti-dsDNA, which remain central to immunologic monitoring. Thus, CBC-derived indices may provide an accessible adjunctive measure for real-world T2T-oriented care and future multicenter validation (4, 5, 21).

Strengths of this study include the use of an actionable clinical endpoint (LLDAS), inexpensive and reproducible CBC−derived biomarkers, a parsimonious and explainable model, comprehensive adjustment for treatment confounders (glucocorticoids and immunosuppressants), and internal validation using bootstrap resampling with calibration and decision curve analyses.

Limitations include its retrospective single−center design, potential unmeasured confounders (e.g., missing autoantibody titers), a modest sample size resulting in an events−per−variable ratio of 8.5 (below the ideal 10), and the lack of external validation. The optimal cutoff values may not generalize directly to other populations, and the observational design precludes causal inference. Future multicenter studies with larger, ethnically diverse cohorts are warranted to validate these findings and calibrate predictive thresholds (22, 23).

In conclusion, after adjustment for major measured confounders, baseline NLR remained independently associated with 1-year LLDAS attainment in patients with SLE. When combined with SLEDAI-2K, it modestly improved discrimination for risk stratification, with bootstrap internal validation suggesting limited overfitting and decision curve analysis supporting potential clinical utility. These findings support CBC-derived indices as feasible, low-cost adjuncts to T2T-oriented management in SLE, while external validation remains necessary before broader clinical application (4–6).

## Data Availability

The original contributions presented in the study are included in the article/Supplementary Material. Further inquiries can be directed to the corresponding author.
