# Correction: Baseline neutrophil-to-lymphocyte ratio combined with SLEDAI predicts lupus low disease activity state at 1 year: a cohort study

**DOI:** 10.3389/fimmu.2026.1915123

**Published:** 2026-07-06

**Authors:** Yadan Zou, Ruohan Yu, Kun Yang, Lina Zhang, Jing Zhang, Ji Li, Ting Long, Yanfeng Zhang, Sheng-Guang Li

**Affiliations:** 1 Department of Rheumatology and Immunology, Peking University International Hospital, Beijing, China; 2 School of Clinical Medicine, Shanxi Medical University, Taiyuan, China

**Keywords:** lupus, Systemic, neutrophil-to-lymphocyte ratio, low disease activity state, cohort Study

There was a mistake in the captions of **Figure 2** and **Figure 3** as published. The captions of the two figures were incorrectly identical. The corrected captions of appear below:

**FIGURE 2** Calibration curves for the three LLDAS prediction models. **(A)** Calibration plot for the NLR‑alone model; **(B)** NLR+SLEDAI‑2K model; **(C)** full six‑variable model.

**FIGURE 3** Decision curve analysis for the three LLDAS prediction models. Net benefit is plotted against threshold probability. The "Treat all" and "Treat none" strategies serve as reference lines. Shaded region (0.20–0.50) indicates the clinically plausible threshold range for initiating treatment escalation.

The original version of this article has been updated.

